# Supplementary material for: Multimodal system for recording individual-level behaviors in songbird groups
Source: PeerJ. 2025 Nov 10;13:e20203. doi: 10.7717/peerj.20203 (PMC12614102; doi:10.7717/peerj.20203)
Supplement: Supplemental Information 1 [file peerj-13-20203-s001.pdf]

Illustrations of the BirdPark Software Suite

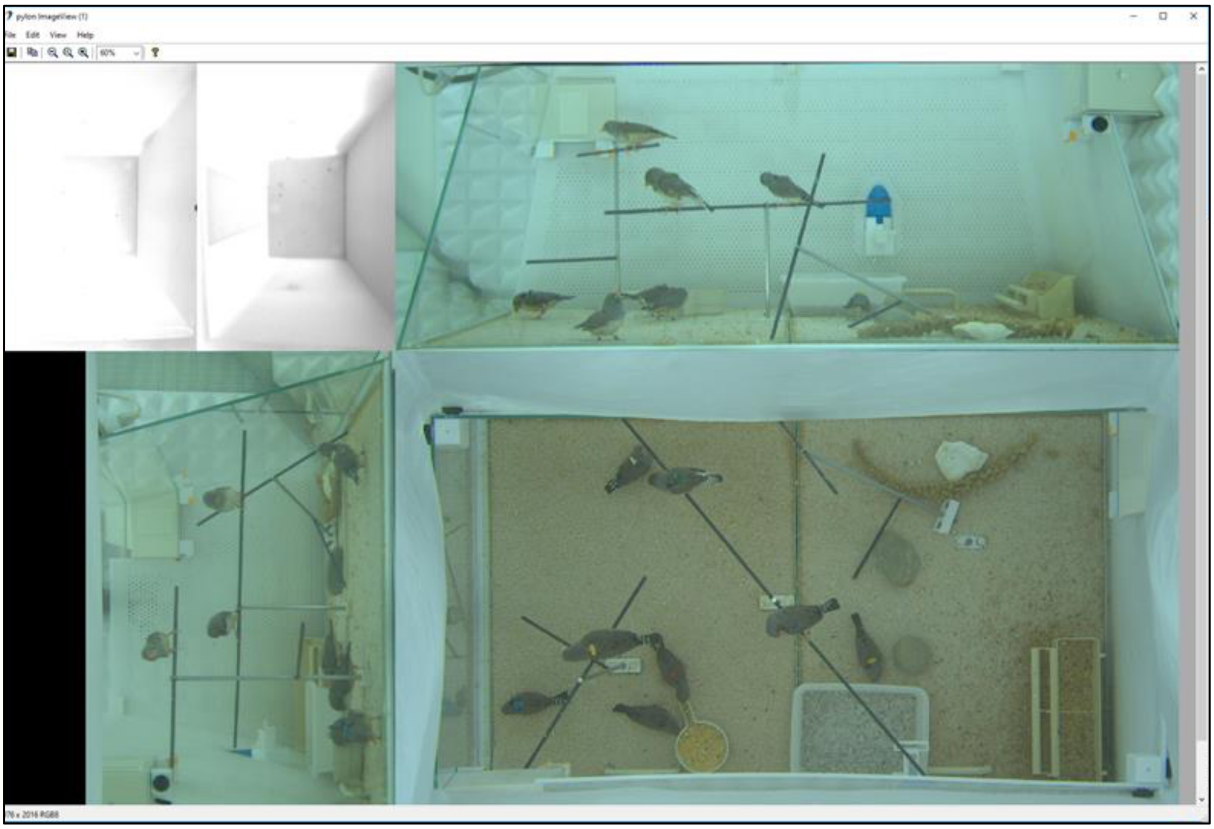

Figure A: Live video monitor of BirdVideo. The monitor displays in real time the composite video frames generated from 3 chamber cameras (side, back, and top views) and from two nest box cameras (top left).

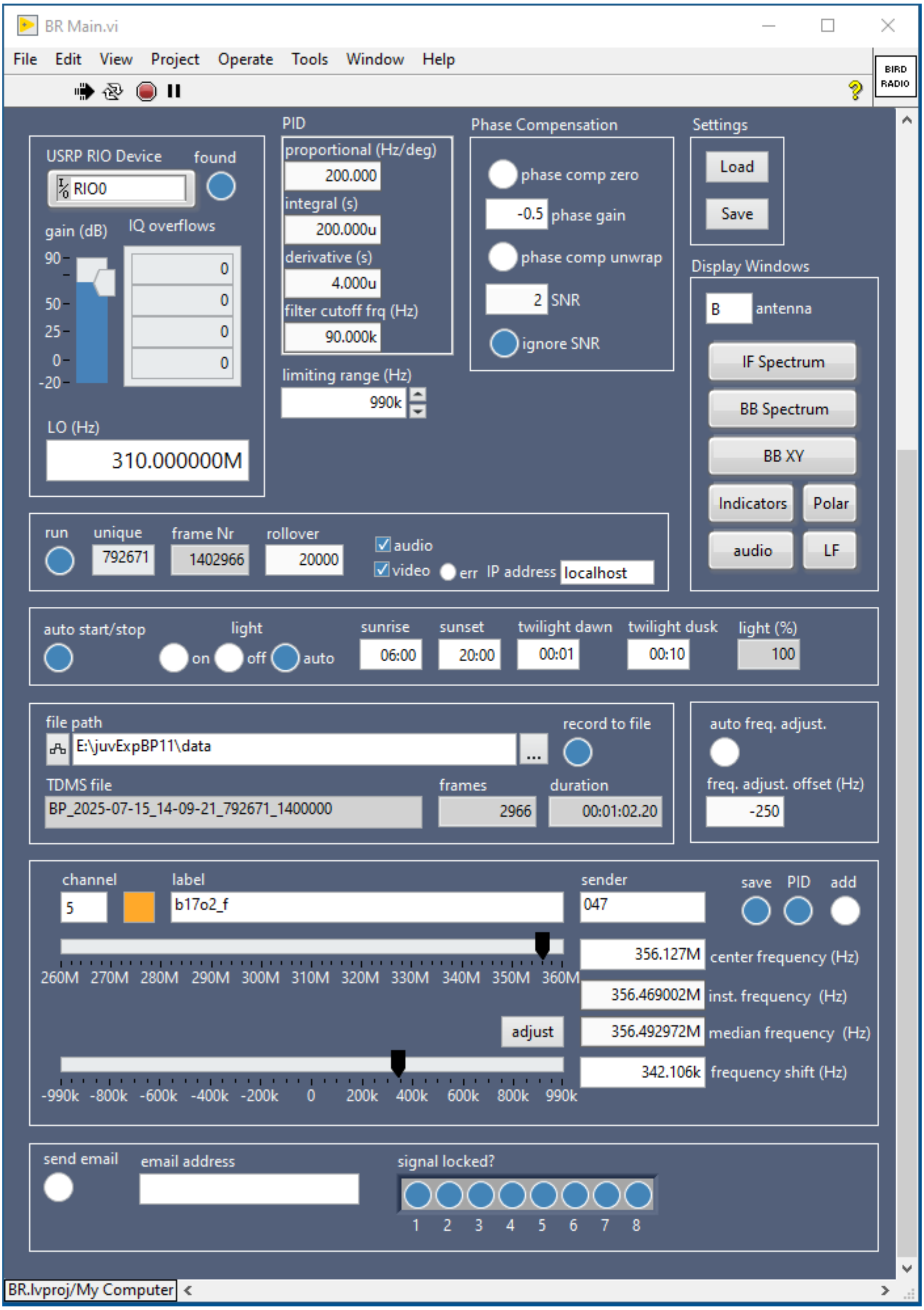

Figure B. Main control panel of BirdRadio. This panel is used to configure the radio preamplifier gains, the eight-channel radio demodulation, the frequency tracking, and the phase compensation. The panel also contains data acquisition start/stop controls.

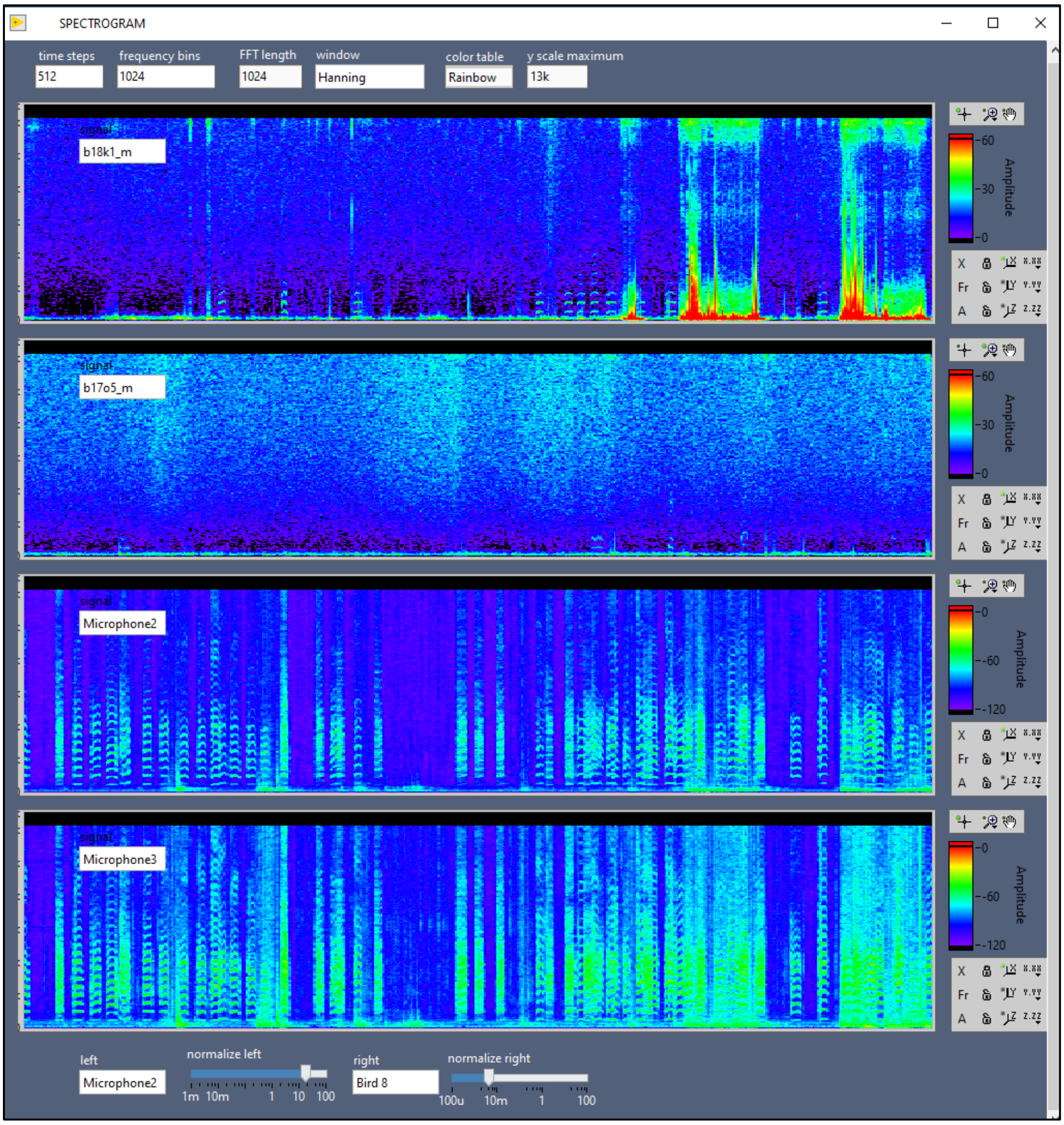

Figure C. Real-time spectrogram display of acceleration signal from bird-mounted transmitters (top two panels) and acoustic signals from wall-mounted microphones (bottom two panels).

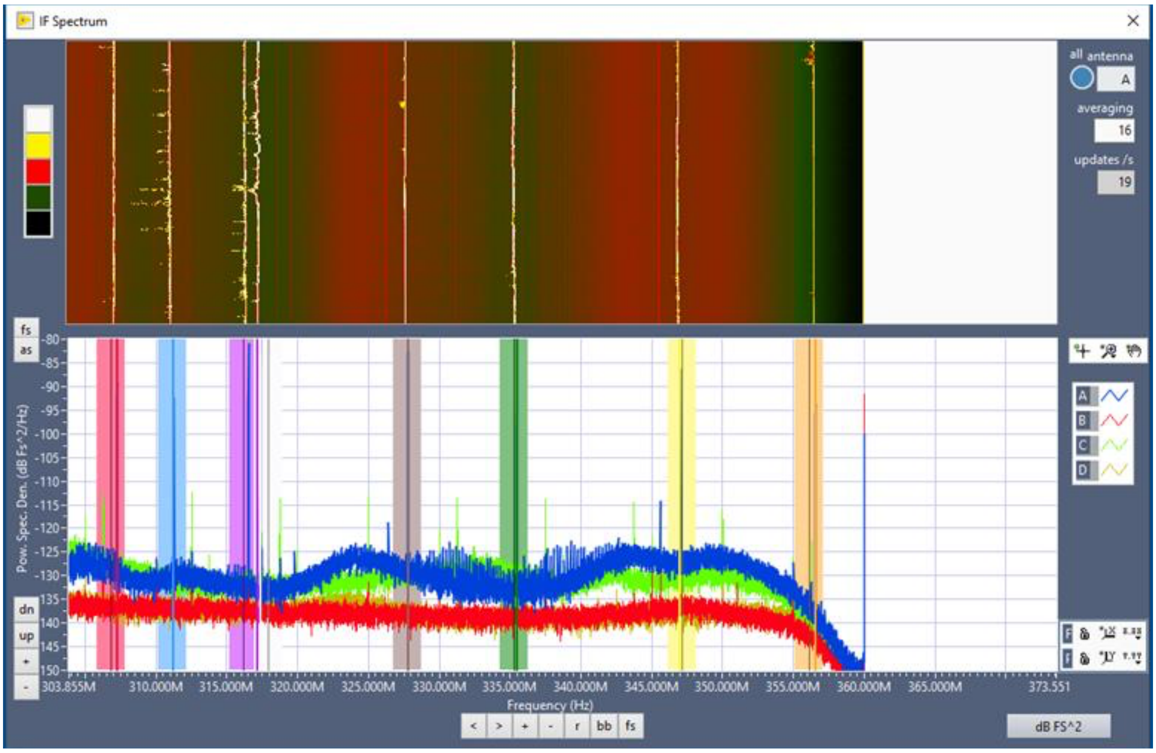

Figure D. Intermediate frequency spectrum. This panel shows the four antenna spectra A-D in distinct colors (blue, red, green, and orange), with eight baseband channels highlighted by colored (including white) vertical bands.

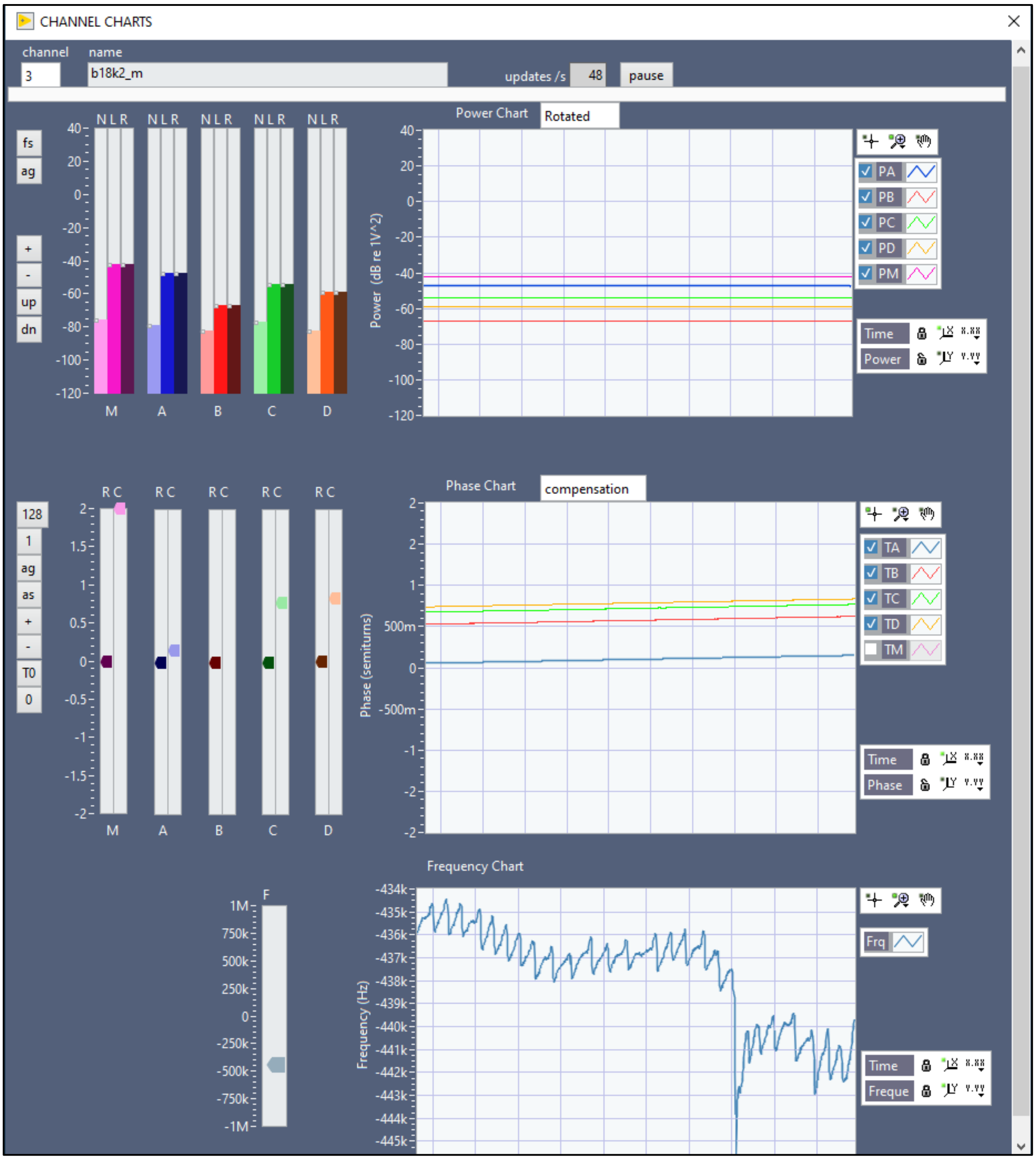

Figure E. Single-channel monitor used for displaying signal amplitudes (top panel), phase relationships (middle panel), and frequency shift (bottom panel).

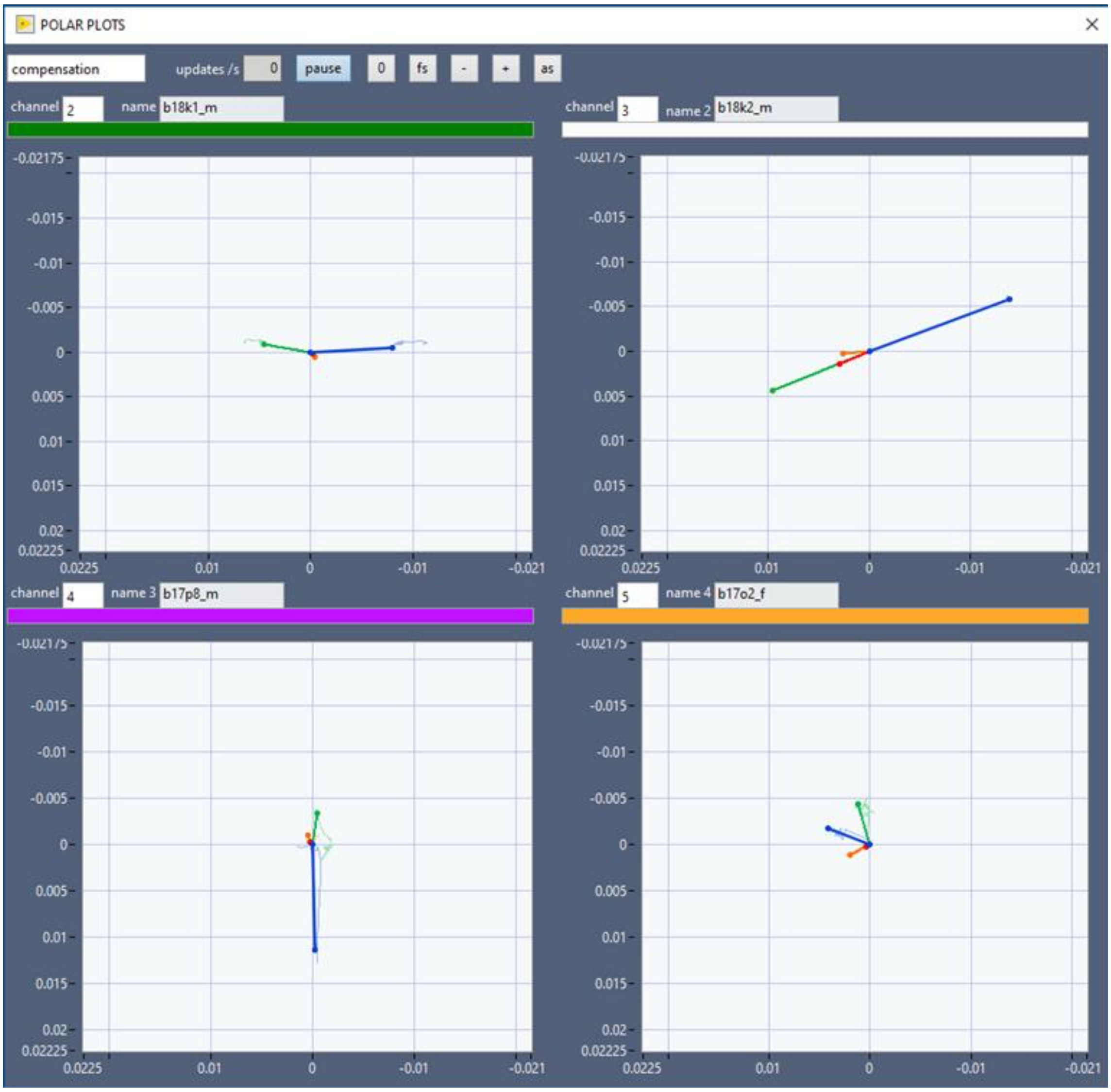

Figure G. Vector plot visualization of the signal amplitudes and compensation phases. The signals from the four antennas are indicated by colors (blue, red, green, and orange). The four panels show different channels (channels $i = 1, \ldots 4$ from top left to bottom right).

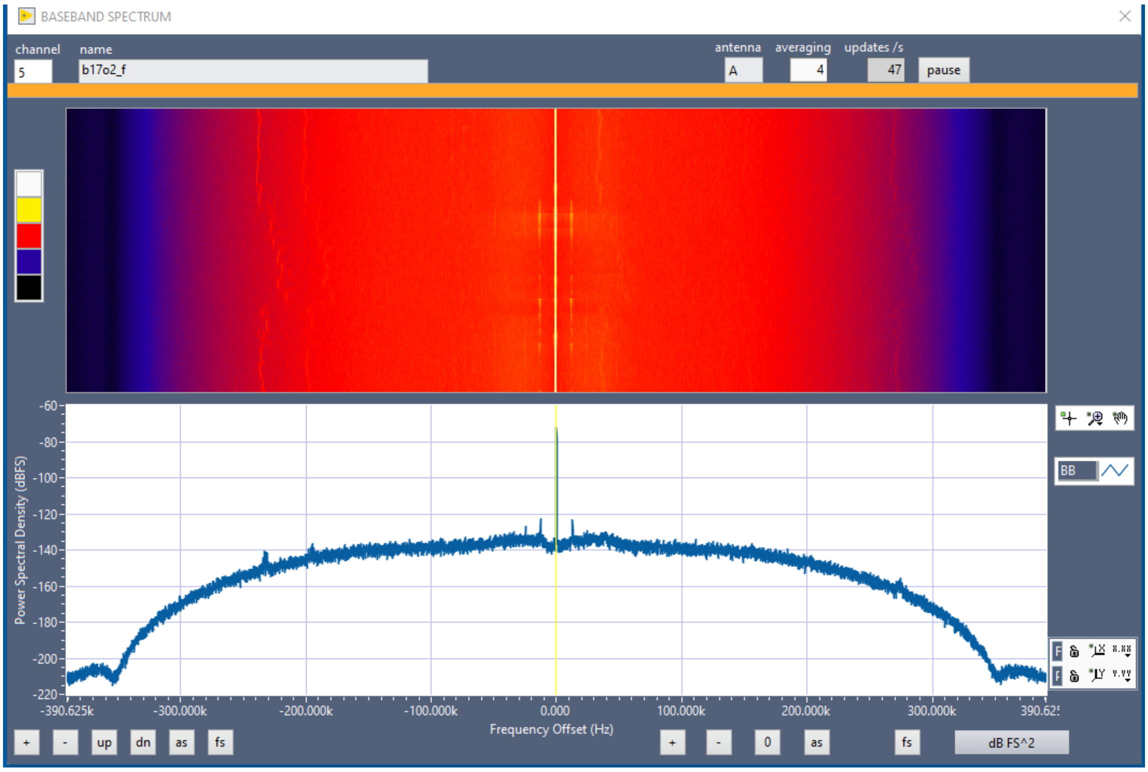

Figure H. Baseband frequency spectrum for channel 5.

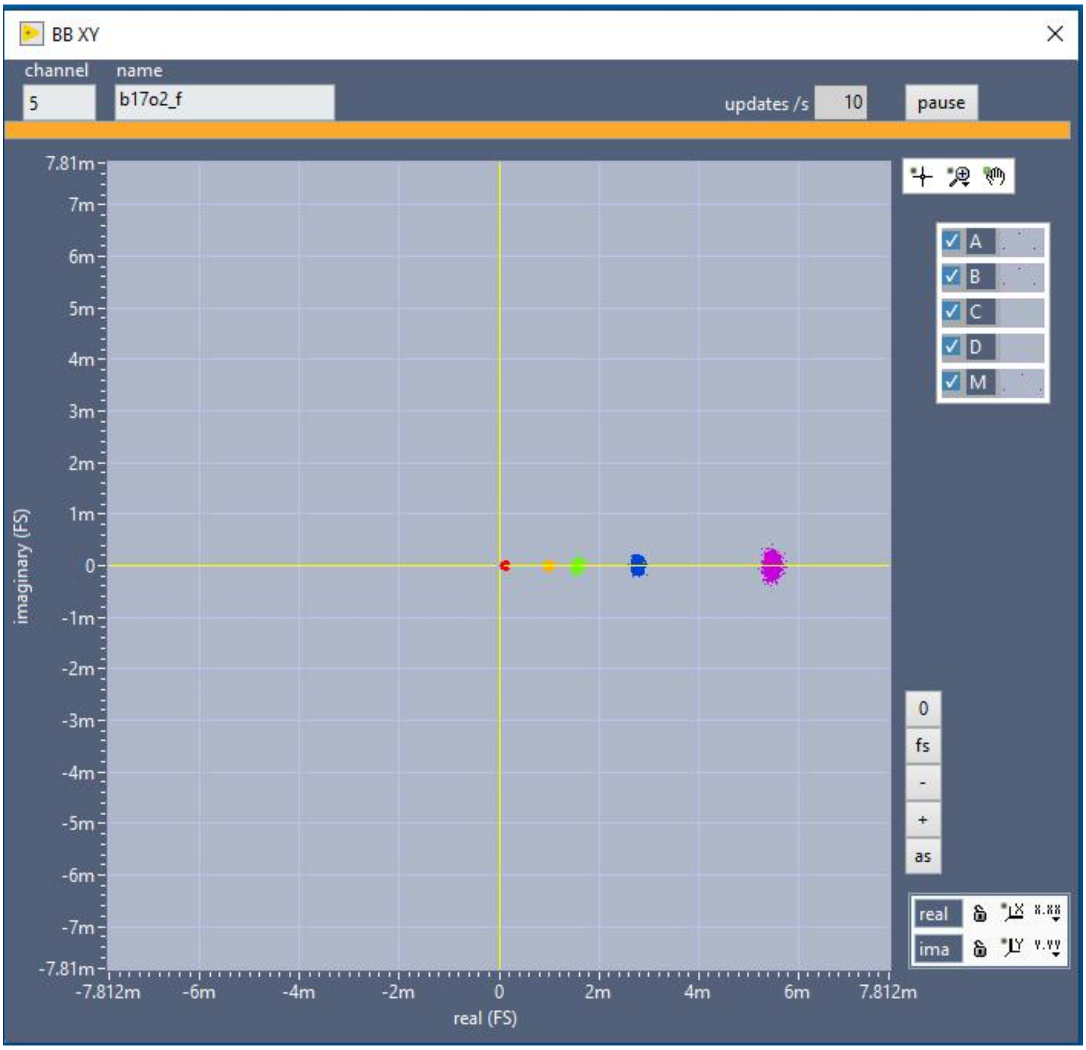

Figure I. Scatter plot of baseband signals associated with a given transmitter (channel 5), displaying data from all four antennas (blue, red, green, and orange) plus the combined main vector (magenta).
